# Etoposide‐induced cancer cell death: roles of mitochondrial VDAC1 and calpain, and resistance mechanisms

**DOI:** 10.1002/1878-0261.13807

**Published:** 2025-02-07

**Authors:** Aditya Karunanithi Nivedita, Varda Shoshan‐Barmatz

**Affiliations:** ^1^ Department of Life Sciences Ben‐Gurion University of the Negev Beer‐Sheva Israel; ^2^ National Institute for Biotechnology in the Negev, Ben‐Gurion University of the Negev Beer‐Sheva Israel

**Keywords:** apoptosis, calpain, etoposide, mitochondria, topoisomerase inhibitors, VDAC1

## Abstract

Etoposide is an inhibitor of DNA topoisomerase II, an enzyme essential for DNA transcription, replication, and chromosome segregation. It is well accepted that etoposide triggers cell death due to DNA damage. Our results indicate that multiple molecular mechanisms contribute to etoposide‐induced apoptosis, including the overexpression of the mitochondrial voltage‐dependent anion channel 1 (VDAC1) and its oligomerization, forming a mega‐channel that releases pro‐apoptotic proteins, thereby activating apoptosis. Etoposide induces C‐terminal truncation of VDAC1 (VDAC1‐ΔC) via the proteolytic actions of calpain‐1 and asparagine endopeptidase (AEP). A calpain‐specific inhibitor effectively prevented etoposide‐induced VDAC1‐ΔC formation, apoptosis, and the nuclear translocation of apoptosis‐inducing factor (AIF). Additionally, etoposide upregulates the expression levels of apoptosis regulators (p53, Bax, p21, AIF) and of the proteases calpain and AEP. Etoposide‐induced apoptosis and VDAC1 truncation are cell‐type dependent and associated with calpain levels and activity. Etoposide‐induced VDAC1‐ΔC formation and apoptosis are tightly linked: as both display similar patterns of concentration‐ and time‐dependence, both are inhibited by calpain and AEP inhibitors, as well as the VDAC1 oligomerization inhibitor VBIT‐4, and are dependent on intracellular Ca^2+^. These findings highlight the complexity of etoposide's actions in different cellular contexts, suggest possible mechanisms of resistance, offer potential biomarkers for guiding etoposide treatment in cancer patients, and propose targeting VDAC1 and calpain as promising therapeutic strategies in cancer therapy.

## Graphical abstract

The complex mode of action of the topoisomerase II inhibitor etoposide in triggering apoptosis involves several mechanisms: overexpression of the mitochondrial protein VDAC1, leading to its oligomerization and formation of a large channel that mediates the release of pro‐apoptotic protein; and overexpression of the apoptosis regulators p53, Bax, and AIF, which increases AEP and calpain levels, catalyzing VDAC1 truncation, and inducing DNA damage.

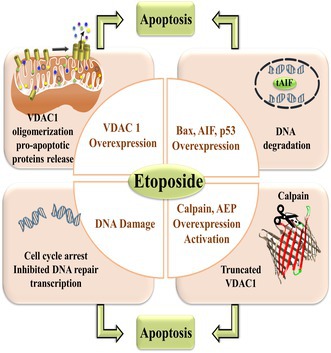

**Abbreviations**
AEP: asparagine endopeptidase
AIF: apoptosis‐inducing factor
BAPTA‐AM: 1,2‐bis(2‐aminophenoxy)ethane‐*N*,*N*,*N*′,*N*′‐tetraacetic acid‐acetoxymethyl ester
EGS: ethylene glycol bis(succinimidyl succinate)
EtBr: ethidium bromide
GBM: glioblastoma multiforme
MDR1: multidrug resistance protein 1
PI: propidium iodide
ROS: reactive oxygen species
VDAC: voltage‐dependent anionic channel

## 1 Introduction

DNA topoisomerases are classified based on their ability to cleave either a single DNA strand (Topo‐I) or a double strand (Topo‐II) [1]. Topo‐I and Topo‐II are nuclear enzymes essential for key DNA metabolism processes, including transcription, replication, chromosome segregation, and polymorphism [2]. Topo‐I and Topo‐II are overexpressed in cancer cells, a making them molecular targets for cancer therapy, owing to their essential role in DNA replication [3].

Inhibitors of Topo‐I and Topo‐II have been shown to trigger both apoptotic and necro‐apoptotic pathways associated with DNA damage and involving the activation of caspases and other pro‐apoptotic factors [4, 5, 6, 7]. Topo‐II inhibitors can be either intercalating compounds, such as anthracyclines and ellipticine, or nonintercalating, such as glycosidic derivatives of podophyllotoxins, teniposide (VM‐26), or etoposide (VP‐16). These inhibitors stabilize the DNA‐Topo‐II complex during DNA replication, leading to DNA damage and subsequent apoptosis [8].

Etoposide is a semisynthetic anticancer agent derived from podophyllotoxin which is produced by *Podophyllum peltatum*. It is widely used in the treatment of lung cancer, testicular cancer, lymphoma, and nonlymphatic leukemia [2].

Here, we propose additional modes of action for Topo‐I and Topo‐II inhibitors in apoptosis induction, involving the mitochondrial protein voltage‐dependent anion channel 1 (VDAC1). VDAC1 is a multi‐functional protein located on the outer mitochondrial membrane (OMM) and is a key regulator of mitochondrial function [9, 10]. Acting as a mitochondrial gatekeeper, VDAC1 controls the metabolic and energetic cross‐talk between the mitochondria and the rest of the cell by mediating the transport of ions, Ca^2+^, ATP, and other metabolites from the intermembrane space (IMS) and cytosol [9, 11, 12, 13].

VDAC1, a critical player in mitochondria‐mediated apoptosis, also mediates the release of pro‐apoptotic proteins, such as cytochrome *c* (Cyto *c*) and apoptosis‐inducing factor (AIF), via a large channel assembled by its oligomerization [11, 13, 14, 15, 16, 17, 18, 19, 20, 21]. Additionally, VDAC1 regulates apoptosis by interacting with both pro‐ and anti‐apoptotic proteins [15, 22]. VDAC1 overexpression and oligomerization are induced by apoptosis stimuli and stress conditions, acting through various mechanisms [14, 16, 19, 23].

VDAC1 located at the OMM, interacts with over 100 proteins associated with metabolism, apoptosis, signal transduction, and other processes with the protein localized in the endoplasmic reticulum (ER), nucleus, cell membrane, and mitochondria. Moreover, VDAC1 is overexpressed in many types of cancers, underscoring to its central role in the pathophysiology of the disease [24].

Mammalian genomes express three VDAC isoforms (VDAC1, 2, and 3), with VDAC1 being the most abundant [9, 25]. VDAC1 is composed of 19 transmembrane β‐strands connected by flexible loops, forming a β‐barrel, with strands β1 and β19 are in a parallel conformation, and the protein includes a 25‐residue‐long N‐terminal region that resides inside the pore [26]. The N‐terminal region is proposed to translocate from the internal pore to the channel surface [27]. Under physiological conditions, VDAC1 exists as monomers and dimers, with a contact site involving β‐strands 1, 2, and 19. However, upon apoptosis induction, VDAC1 dimers undergo conformational changes to assemble into higher oligomeric states, with contact sites also involving β‐strands 8 and 16 [28].

Interestingly, both the N terminus and the C terminus domains of VDAC1 have been proposed to function in apoptosis regulation [16, 29, 30, 31, 32]. It has been shown that deletion of the VDAC1 N‐terminal domain (amino acids 1–26) does not affect cell growth but prevents mitochondrial‐mediated apoptosis. Moreover, the VDAC1 N terminus serves as an interaction site of apoptosis‐regulating proteins of the Bcl‐2 family (i.e., Bax, Bcl‐2, and Bcl‐xL) [16, 27, 33, 34] and hexokinase (HK) [16, 35].

It has been demonstrated that in cultured cells and lung cancer patients, hypoxia induces VDAC1 truncation at the C terminus (VDAC1‐ΔC), which is prevented upon silencing HIF‐1α expression [30, 31]. It is proposed that the formation of VDAC1‐ΔC confers selective protection from apoptosis and allows maintenance of ATP and cell survival under hypoxia conditions [32].

VDAC1 cleavage is mediated by calpains, a family of calcium‐dependent cysteine proteases. The most ubiquitously expressed are μ‐calpain and m‐calpain that can be inhibited by the endogenous inhibitor calpastatin [36]. Activated calpain can activate apoptotic and other signaling pathways [37]. Additionally, hypoxia also induces VDAC1‐ΔC formation by activating the asparagine endopeptidase (AEP or legumain) which is associated with lysosomes.

Here, we demonstrated that treatment of cell with DNA topoisomerase inhibitors, such as etoposide, not only induced apoptosis but also led to the formation of VDAC1‐ΔC, a process dependent on treatment duration, inhibitor concentration, and cell type. This cleavage of VDAC1, associated with apoptosis induction, requires Ca^2+^ and is mediated by calpain and asparagine endopeptidase. The results also demonstrate that etoposide‐induced cell death involves multiple mechanisms, including the upregulation of p53, Bax, AIF, AEP, and calpain. These findings point to the multifaceted etoposide's action and suggest a possible mechanism of etoposide resistance.

## 2 Materials and methods

### 2.1 Materials

Dimethyl sulfoxide (DMSO), etoposide, teniposide, camptothecin, topotecan, ethidium bromide, HEPES, propidium iodide, and 4′, 6‐diamidino‐2‐phenylindole (DAPI) were purchased from Sigma (St. Louis, MO, USA). EGS (ethylene glycol bis (succinimidyl succinate)) and tris were from Fisher Scientific (Loughborough, UK). Annexin V‐(FITC) was obtained from Alexis Biochemicals (Lausen, Switzerland). Fluo‐4‐AM and MitoSOX‐Red were acquired from Invitrogen (Grand Island, NY, USA). BAPTA‐AM (1,2‐Bis(2‐aminophenoxy), ethane‐*N*,*N*,*N*′,*N*′‐tetra acetic acid tetrakis (acetoxymethyl ester)) was from Calbiochem (San Diego, CA, USA). Hank's balanced salts solution (HBSS) without calcium, magnesium, and phenol red was from Biological Industries (Beit Haemek, Israel). Phosphate‐buffered saline (PBS), Dulbecco's modified Eagle's medium (DMEM) and Roswell Park Memorial Institute (RPMI) growth media, normal goat serum (NGS), and the supplements fetal calf serum (FCS), l‐glutamine and penicillin–streptomycin were all obtained from Gibco (Grand Island, NY, USA). siLentFect transfection reagent was obtained from Bio‐Rad (Hercules, CA, USA). Calpain inhibitor I (*N*‐acetyl‐Leu‐Leu‐norleucine) was obtained from Boehringer Ingelheim GmbH (Ingelheim, Germany). Asparagine endopeptidase inhibitor was kindly provided by C. Watts, University of Dundee, Dundee, UK. Nuclear/cytosol fractionation kit was obtained from Biovision (Milpitas, CA, USA).

### 2.2 Cell lines and growth

The cell lines used in this study include HeLa (human cervical adenocarcinoma, RRID: ATCC CCL‐2), SH‐SY5Y (human neuroblastoma, RRID: CRL‐2266), U‐87MG (human glioblastoma, RRID: HTB‐14), PC‐3 (human, prostate; derived from metastatic site: bone, RRID: CRL‐1435), and MDA‐MB‐231 (human, mammary gland/breast; derived from metastatic site: pleural effusion, RRID: HTB‐26) were purchased from the American Type Culture Collection (ATCC, Manassas, VA, USA). Cells were maintained in a humidified atmosphere at 37 °C with 5% CO_2_ in DMEM, or RPMI, supplemented with 10% FCS, 2 mm ‐L‐glutamine, 100 U·mL^−1^ penicillin and 100 μg·mL^−1^ streptomycin, and the cells were routinely tested for mycoplasma contamination.

The cell lines used in this study were obtained from ATCC, and after two passages, they were aliquoted and stored at −80 °C until used. Cells used in this study were between passages 5–15, after the cells were replaced with a freshly thawed sample. Consequently, we did not perform genotyping analysis.

### 2.3 Bax silencing by siRNA transfection

To silence Bax expression, the following human (h)Bax‐siRNA (si‐Bax) were used: sense: 5′‐GGUGCCGGAACUGAUCAGA(dTdT)‐3′ and anti‐sense 5′‐UCUGAUCAGUUCCGGCACC(dTdT)‐3′, as a control, nontargeting, si‐NT: sense 5′‐GCAAACAUCCCAGAGGUAU‐3′, and anti‐sense 5′‐AUACCUCUGGGAUGUUUGC‐3′ were used, both obtained from Genepharma (Suzhou, China). Cells were seeded in 6‐well culture plate (120 000–150 000 cells/well) to 40–60% confluence and transfected with 50 or 70 nm si‐Bax using the siLentFect transfection reagent, according to the manufacturer's instructions.

### 2.4 Cellular Ca^2+^ imaging and analysis

Fluo‐4‐AM was used to monitor changes in cytosolic Ca^2+^ levels. Following the appropriate treatment, cells were harvested, collected (1500 ***g*** for 5 min), and washed with HBSS buffer (5.33 mm KCl, 0.44 mm KH_2_PO_4_, 138 mm NaCl, 4 mm NaHCO_3_, 0.3 mm Na_2_HPO_4_, 5.6 mm glucose, supplemented with 1.8 mm CaCl_2_ (HBSS (+)). Subsequently, approximately 6 × 10^5^ cells·mL^−1^ were incubated with 2.5 μm Fluo‐4 in 200 μL HBSS (+) buffer for 30 min at 37 °C, protected from light. After washing the free Fluo‐4, cells were incubated with 200 μL HBSS (+) buffer, and cellular free Ca^2+^ concentrations were measured immediately by flow cytometer analysis (iCyt Benchtop Cell Analyzer; Sony Biotechnology Inc., San Jose, CA, USA). At least 10 000 events were recorded with positive cells showed a shift to an enhanced level of green fluorescence on the FL1 detector, represented as a histogram, and analyzed by the ec800 flow cytometer software (Sony Biotechnology Inc.). Changes in cellular Ca^2+^ were also monitored in live cells using the high‐content Operetta screening system (Perkin–Elmer, Hamburg, Germany). In each well, 10 fields were imaged using a 20‐fold magnification wide‐field objective, a 520–550 nm excitation filter, and a 560–630 nm emission filter.

### 2.5 Apoptosis assays

#### 2.5.1 Propidium iodide (PI) staining

Cells (2 × 10^5^) were incubated with the desired compound, and after 24, 48, or 70 h, cells were collected (1500 ***g*** for 5 min), washed and resuspended in 200 μL PBS and analyzed for cell death using propidium iodide (PI) staining, added immediately before flow cytometry measurements, followed by flow cytometer analysis.

#### 2.5.2 Annexin V‐FITC/PI staining

Apoptotic cell death was also evaluated using PI and annexin V‐fluorescein isothiocyanate (FITC) (Annexin V–FITC) according to the recommended protocol. Cells were incubation for 15 min, protected from light, then washed once with the binding buffer (10 mm HEPES/NaOH, pH 7.4, 140 mm NaCl, and 2.5 mm CaCl_2_), resuspended in 200 μL binding buffer, and analyzed by flow cytometry. At least 10 000 events were collected, recorded on a dot plot, and analyzed using ec800 flow cytometer software (Sony Biotechnology Inc.).

#### 2.5.3 Acridine orange/ethidium bromide staining

Cell death was additionally assessed using acridine orange (AcOr)/ethidium bromide (EtBr) staining. Cells were centrifuged at 1500 ***g*** for 5 min at 4 °C and resuspended in 25 μL of complete medium. Then, to 2 μL of AcOr/EtBr solution (100 μg·mL^−1^ AcOr and 100 μg·mL^−1^ EtBr in PBS) was added. The stained cells were then visualized by fluorescence microscopy (LX2‐KSP; Olympus, Tokyo, Japan).

### 2.6 Chemical cross‐linking

Cells were treated with etoposide (10–60 μm for 48 or 70 h) and then harvested. The cells were resuspended in PBS (pH 8.3) at a concentration of 2.5–3 mg·mL^−1^ and incubated at 30 °C for 15 min with the indicated concentration of EGS (ethylene glycol bis(succinimidyl succinate)). Cross‐linking was terminated by addition of sample buffer (6% glycerol, 0.6% β‐mercaptoethanol, 1.2% SDS, 100 mm Tris) and heating at 70 °C for 10 min. Cells were then sonicated and subjected to SDS/PAGE followed by immunoblotting using the desired antibodies (Table S1)

### 2.7 Immunofluorescence

SH‐SY5Y cells (2 × 10^5^) were grown on coverslips and treated with etoposide. After 48 h, cells were fixed for 15 min using 4% paraformaldehyde in PBS, followed by rinsing in PBS for 30 min. Permeabilization was achieved by incubating cells in 0.3% Triton X‐100 in PBS (PBST) and blocked with blocking buffer (10% normal goat serum, 1% fatty acid‐free BSA diluted in PBS and 0.1% Triton X‐100) for 2 h. Cells were then probed with the desired primary antibodies (Table S1) diluted in a solution containing 5% normal goat serum, 1% fatty acid‐free BSA in PBS and incubated overnight at 4 °C. The following day, cells were washed three times with PBS and incubated for 2 h at room temperature in the dark with the appropriate fluorescent‐conjugated secondary antibodies (Table S1). Cell imaging was performed using confocal microscopy (Olympus 1X81). Cell nuclei and F‐actin were stained with DAPI for 15 min and with phalloidin for 1 h, respectively. Quantitation of protein levels, as reflected in the staining intensity, was performed across the entire coverslip using imagej software (National Institute of Health, Bethesda, MD, USA).

### 2.8 Measurement of calpain activity

Calpain activity was measured using a calpain activity assay kit (Cat. No.: ab65308; Abcam, Cambridge, UK) following the manufacturer's instructions. Briefly, cells (7 × 10^6^ mL^−1^) were rinsed, detached from the plate, and homogenized in extraction buffer (10 mm Tris/HCl, pH 7.5). The homogenate was centrifuged at 12 000 ***g*** for 10 min, and 50–100 μL of the resulting supernatant (cells lysate) was assayed for activity in a 96‐well plate after protein determination. The calpain substrate Ac‐LLY‐AFC emits blue light (λ_max_ = 400 nm), and following calpain activity, free AFC is released, emitting yellow‐green fluorescence (λ_max_ = 505 nm). Fluorescence was measured using a microplate spectrophotometer at Ex/Em = 400/505 nm using a microplate reader (Tecan Trading, Männedorf, Switzerland).

### 2.9 Protein extraction and quantification

Harvested cells were pelleted by centrifugation at 1500 ***g*** for 10 min at 4 °C. Cells pellet was then resuspended and incubated on ice for 30 min in lysis buffer (50 mm Tris/HCl, pH 7.5, 150 mm NaCl, 1 mm EDTA, 1.5 mm MgCl_2_, 10% glycerol, 1% Triton X‐100), supplemented with a protease inhibitors cocktail (Calbiochem, San Diego, CA, USA). Cell lysate was centrifuged for 10 min at 15 000 ***g*** at 4 °C, and the supernatant was collected and analyzed for protein concentration using the Lowry method. The protein extracts were stored at −80 °C until further analyzed by SDS‐gel electrophoresis and immunoblotting.

### 2.10 Gel electrophoresis and immunoblot analysis

Following SDS/PAGE, gels were either stained with Coomassie Brilliant blue or used for immunoblotting with the selected antibodies. For immunostaining, membranes containing electro‐transferred proteins were initially stained by Ponceau S, washed with H_2_O, and then blocked with 5% nonfat dry milk and 0.1% Tween‐20 in Tris‐buffered saline (TBST, pH 7.8). Subsequently, membranes were incubated with the desired primary antibodies followed by incubation with the appropriate secondary antibodies conjugated with horseradish peroxidase (HRP) (Table S1). Enhanced chemiluminescent substrate (Advantsa, San Jose, CA, USA) was used to detect HRP activity. The relative protein expression level was quantified using the imagej program.

### 2.11 Statistics analysis

The results are presented as the means ± SEM obtained from three or more independent experiments. The level of significance of the differences between the control and treated sample was determined using Student's *t*‐test, conducted with the *t*‐test function in Microsoft Excel. Statistical significance is reported as NS, nonsignificant, *P* > 0.05, *P ≤ 0.05, **P ≤ 0.01, ***P ≤ 0.001, ****P ≤ 0.0001.

## 3 Results

### 3.1 Etoposide‐induced VDAC1 overexpression, oligomerization, and cell death

**Fig. 1:**
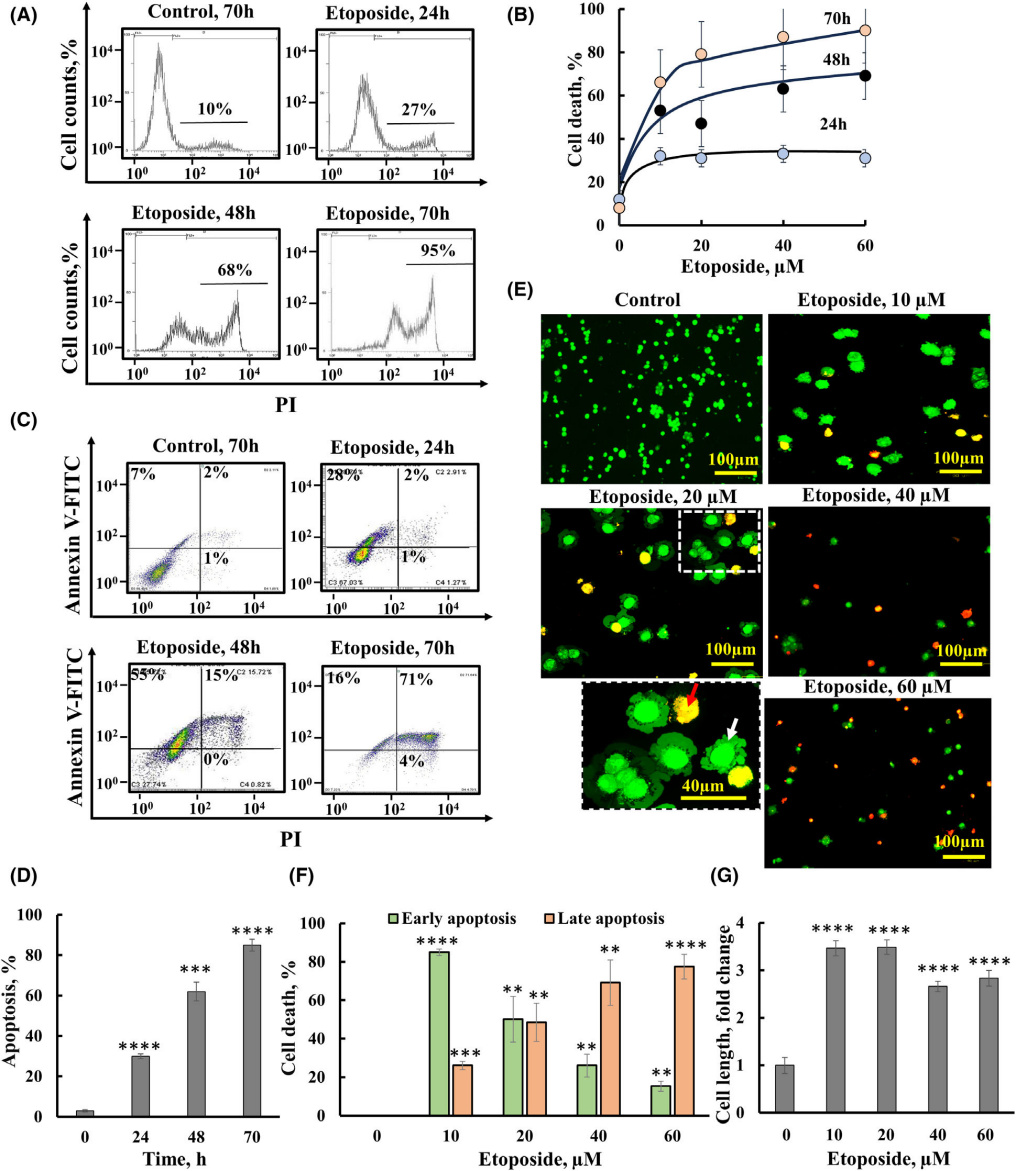
Etoposide induces cell death in SH‐SY5Y cells in a time‐ and concentration‐dependent manner. (A, B) SH‐SY5Y cells were incubated with etoposide (60 μm) for 24, 48, or 70 h, and then, cell death was assayed using propidium iodide (PI) staining and FACS; analysis with representative results (A) and quantification of three independent experiments (B) are shown. The results are the means ± SEM (*n* = 3). (C, D) SH‐SY5Y cells were incubated with etoposide (60 μm) for 24, 48, or 70 h, and apoptosis was assayed using Annexin V‐FITC/PI staining and FACS; analysis with representative results (C) and quantitative analysis of three independent experiments are shown (D). (E, F) SH‐SY5Y cells were incubated with the indicated concentrations of etoposide for 48 h and then stained with acridine orange/ethidium bromide, scale bar = 100 μm. White and red arrows indicate an early apoptotic state and a late apoptotic state, scale bar = 40 μm, respectively, (E), and the cell death quantification is presented in a percentage (F). (G) The average cell length of untreated and etoposide‐treated cells as visualized by acridine orange staining was measured from fluorescent microscope images (*n* = 35 for each group) and presented as fold change relative to etoposide untreated cells. The level of significance of the differences between the control and treated sample was determined using Student's *t*‐test, conducted with the *t*‐test function in Microsoft Excel. Results are the means ± SEM, (*n* = 3). **P ≤ 0.01, ***P ≤ 0.001, ****P ≤ 0.0001. Annexin V‐FITC/PI, PI, and acridine orange/ethidium bromide staining and analysis were performed as described in Section 2.

Etoposide‐induced apoptotic cell death of SH‐SY5Y cells was assayed and assessed using propidium iodide (PI) or PI and Annexin V staining (Fig. 1A–D) or with acridine orange and ethidium bromide (EtBr) staining, which revealing an early apoptotic state represented by membrane blabbing, and a late apoptotic state shown by degraded nuclei stained with EtBr (Fig. 1E,F). Etoposide induced cell death in a concentration‐ and time‐dependent manner, with cell death increasing from 30% to 90% following 24 and 70 h incubations, respectively. Interestingly, etoposide treatment also led to a threefold increase in cell size (Fig. 1E,G).

**Fig. 2:**
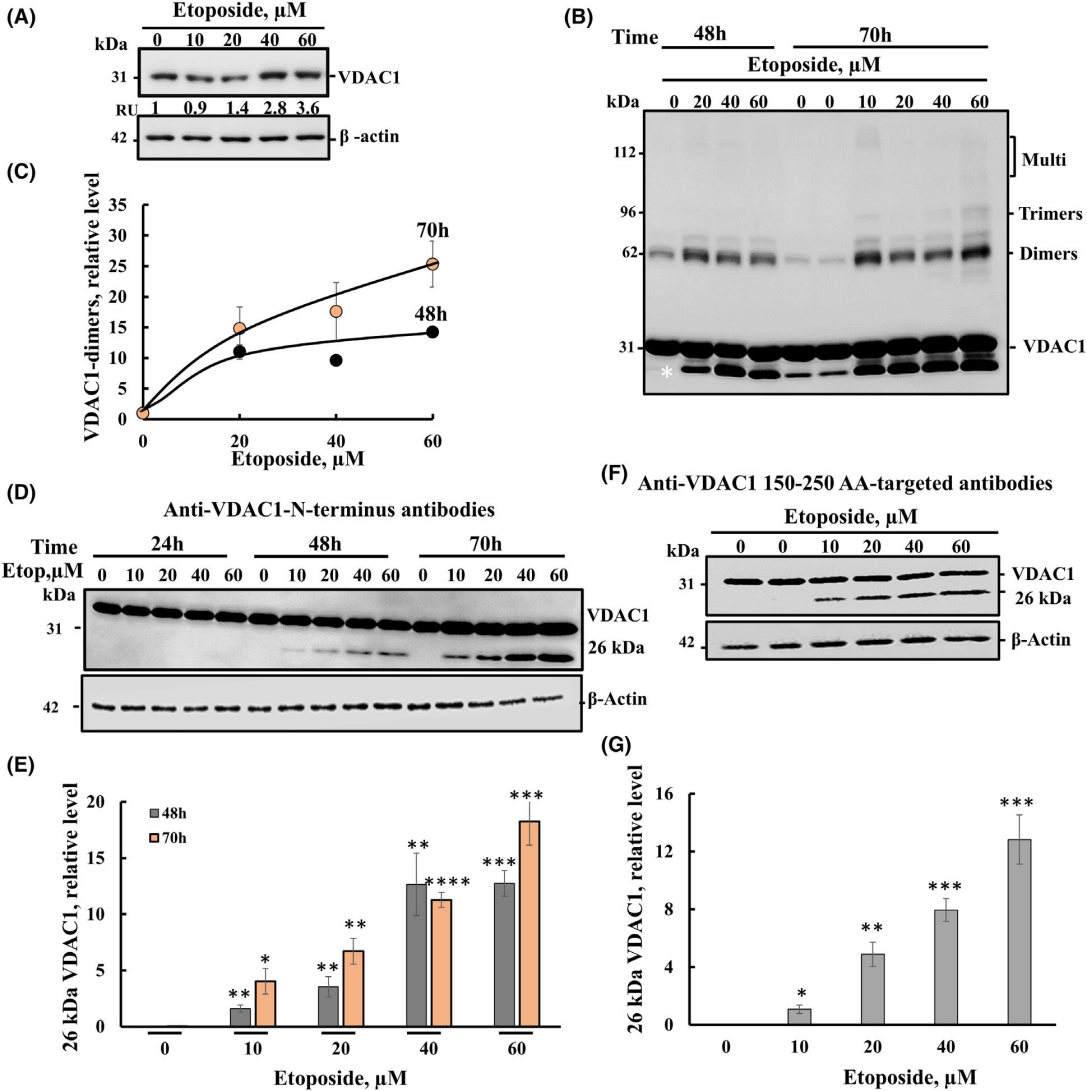
Etoposide induces upregulation of VDAC1 expression and truncation of VDAC1. (A) SH‐SY5Y cells were incubated with or without etoposide (10–60 μm) for 24 h, and VDAC1 expression levels were analyzed by immunoblotting. Quantification of the relative protein levels is presented as relative units (RU) at the bottom of the blot. (B) SH‐SY5Y cells were exposed to etoposide (10–60 μm, 48 or 70 h), washed twice with PBS, and cells (2.5 mg·mL^−1^) were incubated in PBS, pH 8.3 with 250 μm EGS for 15 min at 30 °C. Cell lysates were then subjected to SDS/PAGE and immunoblotted with anti‐VDAC1 antibodies. The positions of VDAC1 monomers, dimers, trimers, and multimers are indicated at the right‐hand side. The VDAC1 band labeled with a * represents an intermolecular cross‐linked VDAC1 [17], which may include also truncated VDAC1. (C) A relative quantification of VDAC1 protein dimers. (D, E) Cells incubated with the indicated concentrations of etoposide (Etop) for 24, 48, or 70 h were subjected to immunoblotting using anti‐VDAC1 antibodies against the N terminus of VDAC1 (D), and the truncated VDAC1 levels were quantified (E). (F, G) SH‐SY5Y cells incubated with the indicated concentrations of etoposide for 48 h were subjected to immunoblotting using antibodies against the 150–250 aa domain of VDAC1 (F), and the relative levels of truncated VDAC1 were quantified (G). The level of significance of the differences between the control and treated sample was determined using Student's *t*‐test, conducted with the *t*‐test function in Microsoft Excel. Results are the means ± SEM (*n* = 3). *P ≤ 0.05, **P ≤ 0.01, ***P ≤ 0.001, ****P ≤ 0.0001.

Etoposide induced in SH‐SY5Y cells VDAC1 overexpression (about fourfold) and VDAC1 oligomerization in a concentration‐dependent manner (Fig. 2A–C) Cells treated with etoposide resulted in VDAC1 oligomerization, as revealed using the cross‐linker EGS, that resulted in the appearance of VDAC1 dimers, trimers, and higher oligomers (Fig. 2B,C). These findings suggest that etoposide, like other apoptosis stimuli [14, 16, 19, 23], induces overexpression of VDAC1, shifting the equilibrium toward the VDAC1 oligomeric state, that allowing the release of pro‐apoptotic proteins that lead to cell death.

During the characterization of etoposide‐induced VDAC1 overexpression, oligomerization, and apoptosis, we observed induction of VDAC1 truncation (Fig. 2D–G). SHSY‐5Y cells incubated with different concentrations of etoposide for 48 or 70 h, but not 24 h, showed the appearance of a protein band corresponding to 26 kDa, as detected using antibodies against the N‐terminal domain (produced against human VDAC1 1–100 amino acids) (Fig. 2D,E). The levels of the truncated VDAC1 were increased with the incubation time, being higher after 70 h incubation compared to its level at 48 h. Truncated VDAC1 was also detected using antibodies produced against an internal VDAC1 domain (corresponding to human VDAC1 amino acids 150–250) (Fig. 2F,G). Given the detection of truncated VDAC1 with both antibodies, we concluded that VDAC1 was truncated at the C terminus (VDAC1‐ΔC).

### 3.2 Etoposide increased mitochondrial reactive oxygen species and cellular Ca^2+^ levels

**Fig. 3:**
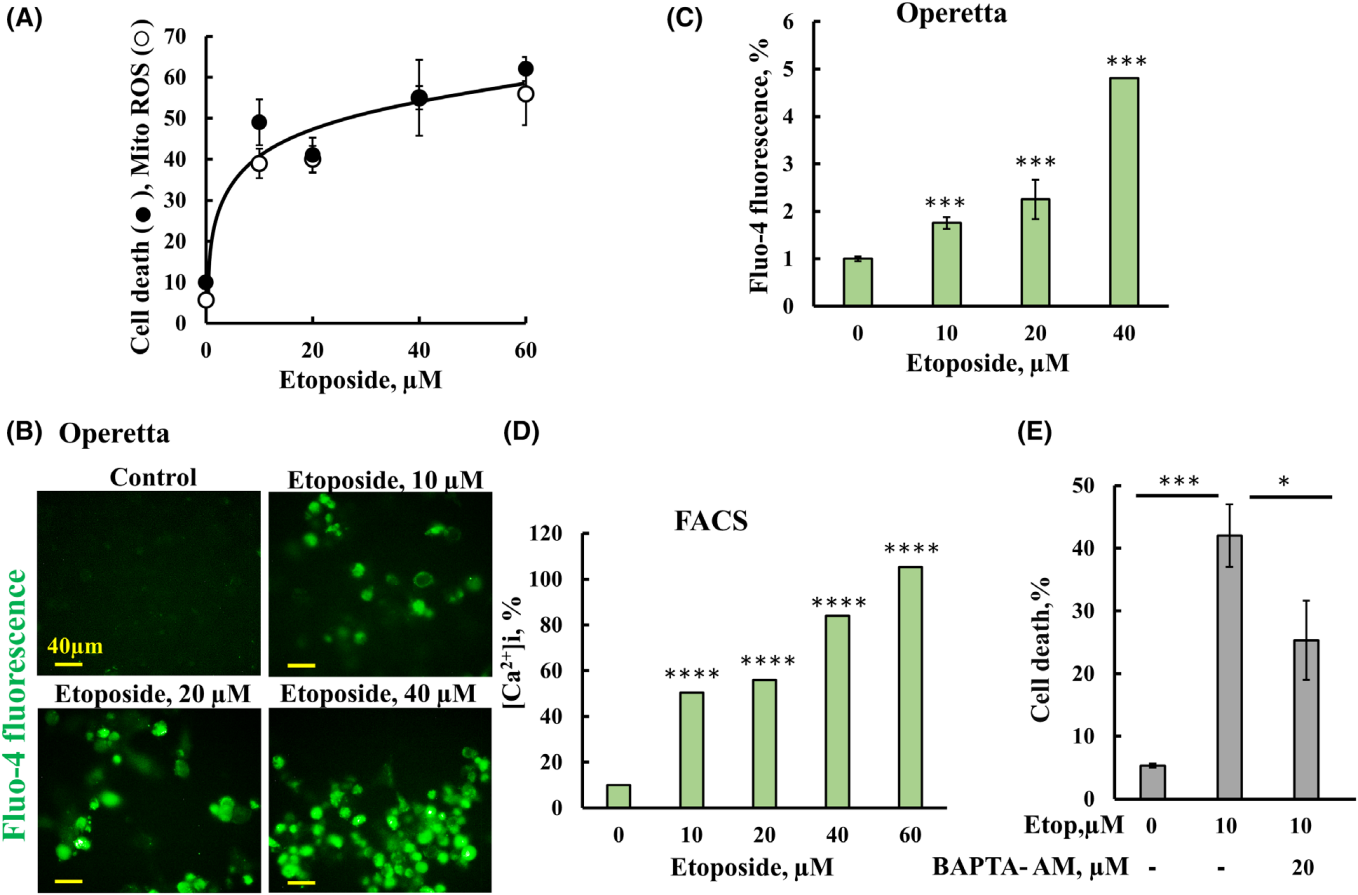
Etoposide induces elevated mitochondrial ROS and cytosolic Ca^2+^. (A) SH‐SY5Y cells were treated for 48 h with the indicated concentrations of etoposide, followed by incubation with Mito‐SOX for mitochondrial ROS analysis or subjected to PI (Propidium Iodide) staining for cell death analysis using FACS. (B) SH‐SY5Y cells were seeded on black cell carrier 96‐well plates, and after 24 h, cells were treated with the indicated concentration of etoposide for 48 h, followed by incubation with Fluo‐4, and cell fluorescence imaging was recorded using the Operetta, scale bar = 40 μm (B) and quantified (C). (D) Changes in cytosolic Ca^2+^ were also analyzed with cells treated for 48 h with etoposide at the indicated concentrations, followed by incubation with Fluo‐4 and FACS analysis. (E) SH‐SY5Y cells were pre‐incubated with or without BAPTA‐AM (20 μm, 1 h), then incubated 48 h with etoposide (10 μm), and assayed for cell death using PI staining and FACS analysis. The level of significance of the differences between the control and treated sample was determined using Student's *t*‐test, conducted with the *t*‐test function in Microsoft Excel. Results are the means ± SEM (*n* = 3). *P ≤ 0.05, ***P ≤ 0.001, ****P ≤ 0.0001.

Apoptosis induction also resulted in increased production of reactive oxygen species (ROS). Mitochondria are the major source of ROS which, when not eliminated, are released to the cytosol via VDAC1 [38], thereby modifying DNA, lipids, and proteins, affecting cell survival, and inducing VDAC1 overexpression [38]. Similar to other apoptosis‐inducing, such as inorganic arsenic compounds [39] and doxorubicin [40], etoposide also increased ROS generation which is highly correlated with cell death (Fig. 3A).

In previous studies [30, 31], the hypoxia‐induced VDAC1‐ΔC was proposed to result from calpain activity. Since calpains are calcium‐dependent proteases, we tested the effect of etoposide on the cellular calcium [Ca^2+^]i levels. Etoposide increased [Ca^2+^]i levels, as monitored by Fluo‐4 and cell fluorescence recording using the high‐content imaging system Operetta (Fig. 3B,C) and by flow cytometer analysis (Fig. 3D).

**Fig. 4:**
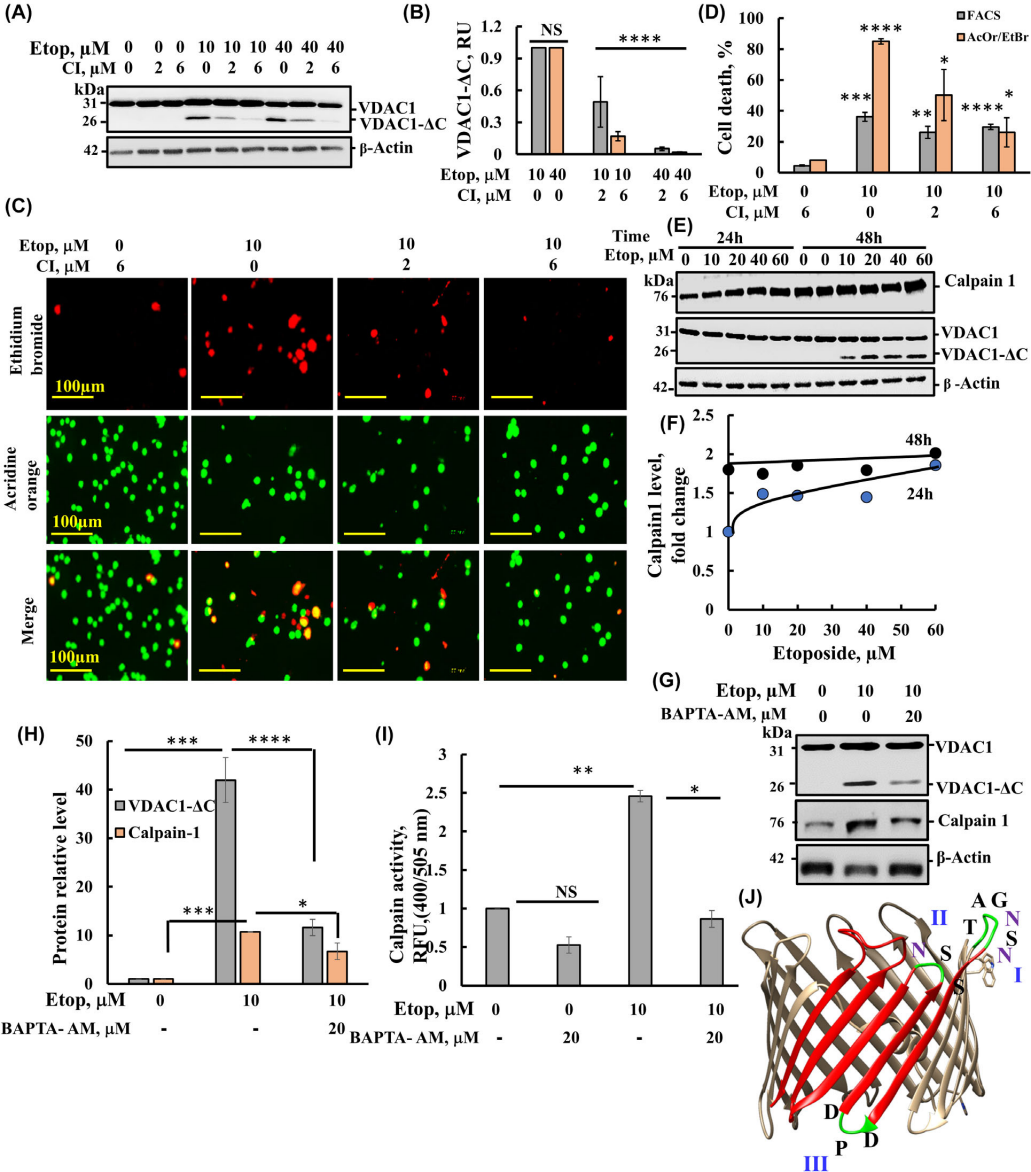
Etoposide‐induced overexpression of calpain and inhibition of VDAC1 truncation by the calpain inhibitor. (A, B) SH‐SY5Y cells pre‐incubated with calpain inhibitor (CI, 2 or 6 μm, 1 h), were incubated with the indicated concentrations of etoposide for 48 h, and the levels VDAC1‐ΔC formation were analyzed by immunoblotting (A) and quantification (B). (C, D) SH‐SY5Y cells pre‐incubated with CI (2 or 6 μm, 1 h), were incubated for 48 h with or without etoposide (10 μm), and assayed for cell death using acridine orange/ethidium bromide staining by microscope (scale bar = 100 μm) (C) and by PI staining and FACS analysis (D). (E, F) SH‐SY5Y cells incubated with the indicated concentrations of etoposide for 24 h and 48 h were analyzed by immunoblotting with specific antibodies against VDAC1 and calpain‐1 (E). Quantification of the relative calpain expression levels is shown (F). (G) SH‐SY5Y cells pre‐incubated with or without BAPTA‐AM (20 μm, 1 h), were incubated with etoposide (10 μm, 48 h), analyzed for VDAC1, VDAC1‐ΔC, and calpain‐1 levels by immunoblotting using anti‐VDAC1 and calpain‐1 antibodies, and relative levels were quantified (H). (I) SH‐SY5Y cells pre‐incubated with BAPTA‐AM (20 μm, 1 h), were incubated with or without etoposide (10 μm, 16 h), and assayed for calpain activity. (J) The structure used for modeling the VDAC1 membrane topology was derived from PDB ID: 3EMN, presenting the proposed calpain and AEP cleavage sites in the loop (in black) and asparagine residues (in purple) were visualized using ucsf chimera software. The significance of the differences between the control and treated samples was determined using Student's *t*‐test, conducted with the *t*‐test function in Microsoft Excel. Results are the mean ± SD, (*n* = 3). *P ≤ 0.05, **P ≤ 0.01, ***P ≤ 0.001, ****P ≤ 0.0001.

To evaluate the requirement of Ca^2+^ for VDAC1‐ΔC formation, cells were pre‐incubated with the cell‐permeable Ca^2+^ chelating reagent, BAPTA‐AM. Pre‐incubation with BAPTA‐AM significantly inhibited both etoposide‐induced cell death, VDAC1‐ΔC formation, and calpain‐1 expression (Figs 3E and 4G,H). The effects of etoposide and BAPTA‐AM on calpain activity was assayed in lysates of SH‐SY5Y cells pre‐incubated with etoposide in the absence or the presence of BAPTA‐AM (Fig. 4I). The results showed that etoposide increased calpain activity by about 2.5‐fold, but not when cells were also pretreated with BAPTA (Fig. 4G,H). The reduced activity in cell lysate treated with both etoposide and BAPTA in agreement with increasing its expression level (Fig. 4G,H). These findings indicate the requirement of Ca^2+^ for both VDAC1‐ΔC formation and calpain activity.

Interestingly, VBIT‐4, which decreases intracellular calcium induced by apoptotic inducers [41], also prevented VDAC1‐ΔC formation (Fig. S1G).

### 3.3 Calpain and asparagine endopeptidase mediate VDAC1‐ΔC formation

Calpains are a family of Ca^2+^‐dependent cysteine proteases that catalyze the proteolysis of a large number of specific proteins. Mitochondrial μ‐calpain has been shown to cleave VDAC1 [42].

To determine whether calpain is involved in etoposide‐induced VDAC1 truncation, we examined the effects of a calpain‐specific inhibitor (CI) on the formation of VDAC1‐ΔC and cell death triggered by etoposide (Fig. 4A–D). The CI effectively inhibited the formation of VDAC1‐ΔC, induced by etoposide, achieving complete inhibition obtained at 6 μm (Fig. 4A,B). The effect of CI on cell death was analyzed by PI staining and FACS analysis, and with AcOr and EtBr staining, demonstrating that etoposide‐induced cell death was inhibited by CI in a concentration‐dependent manner (Fig. 4C,D).

These finding suggest that calpain‐1 mediates VDAC1‐ΔC formation, presumably activated by an etoposide induced increase in cytosolic Ca^2+^. Interestingly, etoposide treatment also resulted in elevated calpain expression levels (Fig. 4E,F). The results show that after a 24‐h incubation, etoposide increased calpain‐1 expression levels in a concentration‐dependent manner. However, following a 48‐h incubation, a similar increase in calpain‐1 expression was observed both with and without etoposide treatment (control) (Fig. 4E,F).

Considering VDAC1 membrane topology and the subcellular localization of calpains a in the IMS and the cytosol, along with the common motif for calpain cleavage sites, we predicted three potential calpain cleavage sites (I, II, and III) on VDAC1 (Fig. 4J). Any of the three potential cleavage sites in VDAC1 would yield cleavage products with a very similar molecular mass (26 kDa).

Asparagine endopeptidase is a cysteine endopeptidase that specifically cleaves peptide bonds in asparaginyl residues (Asn/Asp (Asx)) and is associated with lysosomes [6, 43]. Previous studies have shown that also AEP, under hypoxic conditions, produces VDAC1‐ΔC [30, 31]. To assess the role of AEP in etoposide‐induced VDAC1‐ΔC formation, we tested the effect of the AEP‐specific inhibitor MV026630 (AEP‐I) on etoposide‐induced VDAC1‐ΔC. The results demonstrated that AEP‐I strongly inhibits VDAC1‐ΔC formation induced by etoposide (Fig. S1C).

Asparagine endopeptidase activation is autocatalytic, and a bimolecular reaction requires sequential removal of a short 8‐residues at the N‐terminal and 110‐residues at the C‐terminal of the pro‐AEP cleavages, leading to the final mature and active lysosomal enzyme [44]. We observed that etoposide induced an increase in pro‐AEP (56 kDa) in a concentration‐ and incubation time‐dependent manner (Fig. S1B,C). Although the levels of the mature‐1 AEP (46 kDa), representing auto cleavage at the N terminus, showed slight increase, the levels of the mature‐2 (36 kDa) protein were increased after etoposide treatment for 24 h (Fig. S1B–E)). Interestingly, the levels of mature‐2‐AEP were highly increased with and without etoposide treatment (Fig. S1B,E), resembling the observations for the expression calpain‐1 and AIF (Fig. 4E). Since AEP activity is not Ca^2+^‐dependent but its promoter is activated by Ca^2+^ [45], this suggests that its increased expression may result from the etoposide‐induced increase in [Ca^2+^] (Fig. 3).

### 3.4 Etoposide‐induced calpain‐mediated AIF activation and translocation

**Fig. 5:**
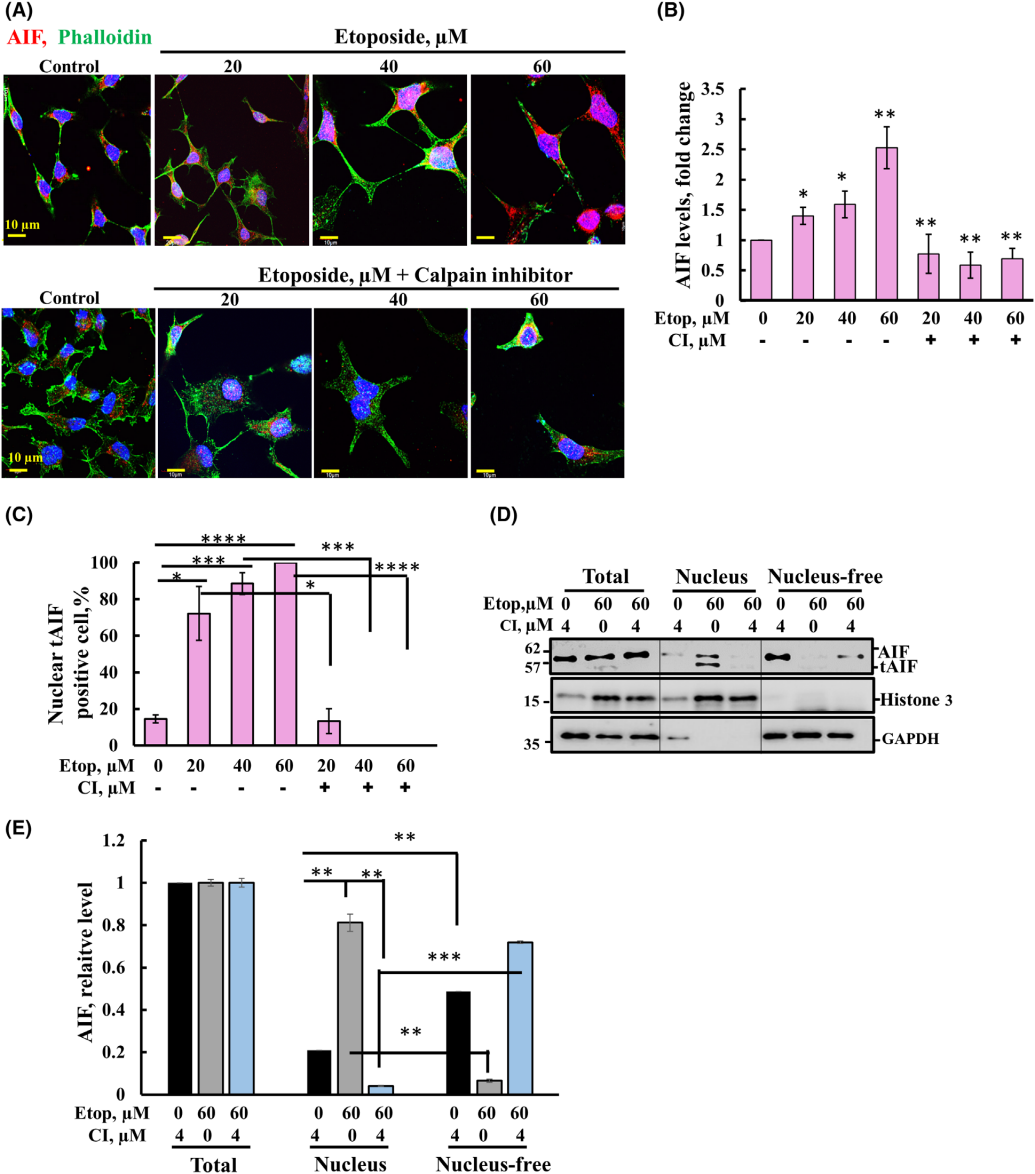
Etoposide‐induced calpain‐mediated AIF activation and translocation. (A) Representative images of cells immune fluorescently co‐stained with anti‐AIF and phalloidin (for actin) and DAPI (for the nucleus) of SH‐SY5Y cells incubated with the indicated etoposide concentrations in the absence or first pre‐incubated with CI (calpain Inhibitor) (4 μm, 1 h) followed by 48 h, scale bar = 10 μm. (B, C) Quantitative analysis of AIF levels with the indicated *P* values were obtained relative to the samples treated with the same etoposide concentration and in the absence of the CI (Calpain Inhibitor) and its nuclear localization presented as the percentage of cells with nuclear AIF. (D) For AIF subcellular localization, SH‐SY5Y cells were subjected to nuclear and cytosol fractions using a nuclear/cytosol fractionation kit (Biovision, Milpitas, CA, USA), according to the manufacturer's instructions. The total represents the cells subjected to the extraction buffer treatment before centrifugation. The cytosolic and nuclear fractions represent the supernatant and pellet, respectively, obtained following centrifugation (16 000 ***g***, 10 min). The pellet was resuspended in the original volume. Identical volumes of the above fractions were subjected to immunoblotting using specific antibodies against AIF and tAIF, histone 3 (H3, marker for nuclei), and GAPDH (cytosolic). AIF relative levels in the different fractions were quantified, and the significant of the differences between the cytosolic and nuclear fractions are presented (E). Phalloidin staining, nuclear/cytosol fractionation, and immunoblotting were carried out as described in Section 2. The significance of the differences between the control and treated sample was determined using Student's *t*‐test, conducted with the *t*‐test function in Microsoft Excel. Results are the means ± SEM (*n* = 3). *P ≤ 0.05, **P ≤ 0.01, ***P ≤ 0.001, ****P ≤ 0.0001. NS, nonsignificant.

Given that calpain mediates cleavage of AIF before its release from the mitochondria [46], we tested whether etoposide also activates calpain‐mediated AIF activation and subsequent translocation to the nucleus (Fig. 5A–E). Immunofluorescence staining for AIF revealed increased AIF expression levels in etoposide‐treated cells in a concentration‐dependent manner that was inhibited by CI (Fig. 5A,B). Similarly, etoposide treatment resulted in enhanced AIF nuclear localization that is inhibited by CI (Fig. 4C–E). To further demonstrate AIF translocation to the nucleus, we used cell fractionization to assess cytosolic and nuclear fractions. The distribution of AIF between the cytosolic/mitochondrial and nuclear fractions clearly shows that upon etoposide treatment, AIF levels were increased about fourfold in the nucleus, but not in CI‐treated cells (Fig. 5D,E). This suggests that etoposide induced AIF translocation to the nucleus involves calpain activity. Additionally, an etoposide‐induced increase in cell size was also visualized by phalloidin‐stained actin (Fig. S2D,E).

These results suggest that etoposide, through the activation of calpain mediating AIF maturation and its nuclear translocation, and thus also activates AIF‐mediated cell death.

### 3.5 Etoposide upregulates the expression of apoptosis‐related proteins

**Fig. 6:**
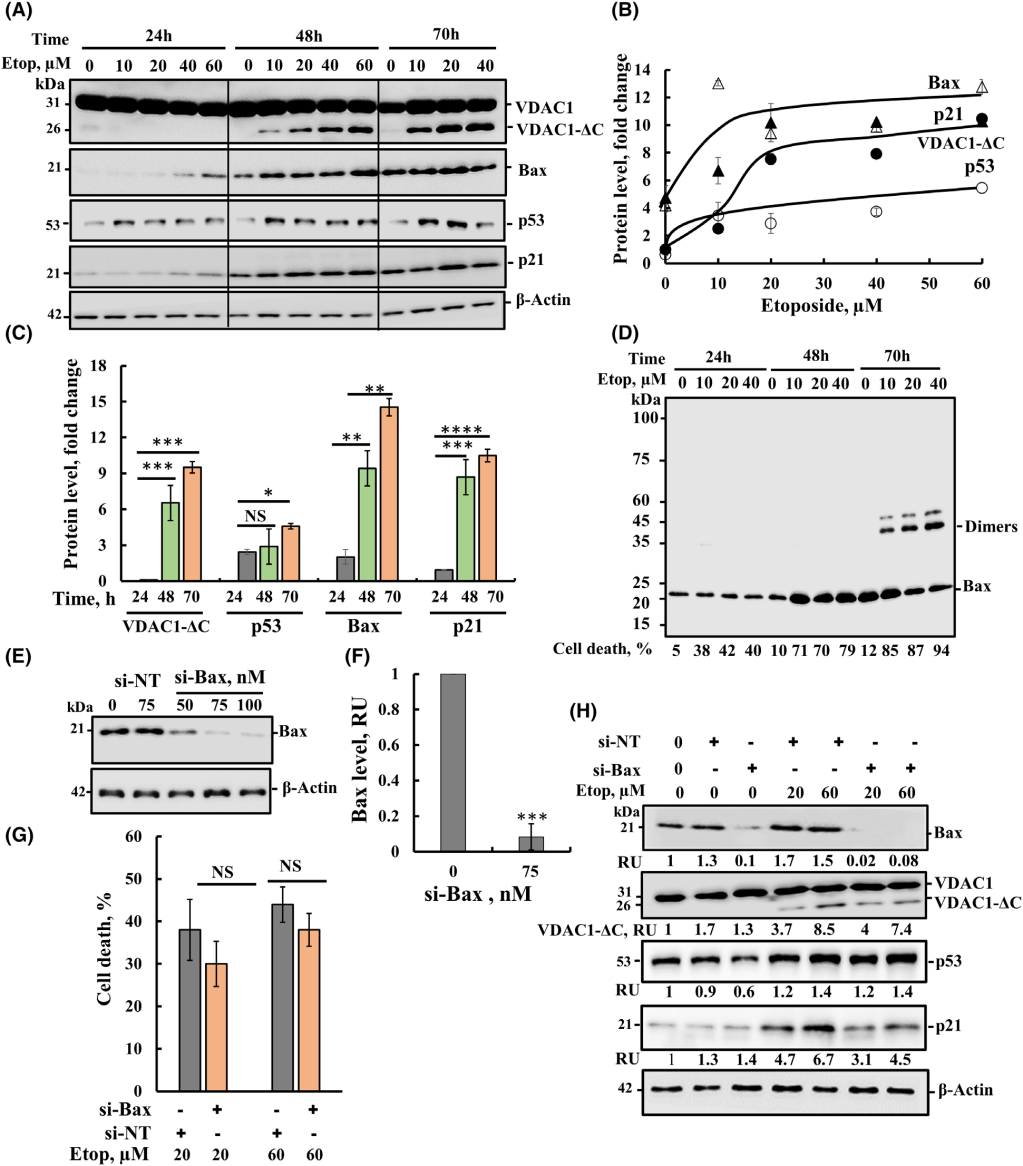
Etoposide upregulates the expression of apoptosis‐related proteins. (A–C) SH‐SY5Y cells were incubated with the indicated concentrations of etoposide for 24, 48, or 70 h, then subjected to immunoblotting using specific antibodies against VDAC1, Bax, p53, p21 or β‐actin (A), and their expression levels following incubation with different concentrations of etoposide for 48 h (B) or following incubation with etoposide (20 μm) for 24, 48, and 70 h (C) are presented as fold change with the indicated *P* values were obtained relative to the samples treated for 24 h (D) SH‐SY5Y cells were exposed to etoposide (10–40 μm, 24, 48, and 70 h), washed twice with PBS, and cells (2.5 mg·mL^−1^) were incubated at PBS, pH 8.3 with the 250 μm EGS for 15 min at 30 °C, and then subjected to SDS/PAGE and immunoblotting using anti‐Bax antibodies. Cell death was analyzed in parallel using PI staining and FACS analysis and is presented at the bottom. (E, F) SH‐SY5Y cells were transfected for 48 h with the indicated concentrations of si‐NT or si‐Bax using siLentFect transfection reagent, and Bax expression levels were analyzed by immunostaining using anti‐Bax antibodies. Bax levels were quantified and are presented as relative units (RU) (F). β‐Actin was used as a loading control. (G, H) Cells were transfected with si‐NT (nontargeted) (75 nm) or si‐Bax (75 nm), and 48 h post‐transfection, cells were treated (48 h) with 20 or 60 μm of etoposide, analyzed for cell death using PI staining and FACS analysis (G) and for the expression levels of Bax, VDAC1 and VDAC1‐ΔC, p53, p21 or β‐actin (H). The intensity of the bands was quantified using imagej software and presented at the bottom of each blot in relative units (RUs). Immunoblotting, si‐BAX cell treatment, cross‐linking, and PI staining were performed as described in Section 2. The significance of the differences between the control and treated sample was determined using Student's *t*‐test, conducted with the *t*‐test function in Microsoft Excel. Results are the means ± SEM (*n* = 3). *P ≤ 0.05, **P ≤ 0.01, ***P ≤ 0.001, ****P ≤ 0.0001. NS, nonsignificant.

Etoposide treatment that resulted in VDAC1 overexpression, oligomerization, and truncation also induced the overexpression of the pro‐apoptotic protein Bax and the tumor suppressor p53 in a time‐ and concentration‐dependent manner (Fig. 6A–C). To assess active p53, the expression of p21 protein, an inhibitor of cyclin‐dependent kinases, and a downstream target of p53, was monitored. The results revealed that upon etoposide treatment an increase in p21 expression levels was obtained (Fig. 6A–C). Following a 24‐h incubation with etoposide, the levels of Bax and p21 were increased as a function of etoposide concentration. However, cells growing for 70 h showed a similar increase in the expression levels of these proteins, with and without etoposide treatment (Fig. 6A). This is similar to the results obtained with calpain (Fig. 4E,F).

To evaluate the relationship between etoposide‐induced cell death and Bax overexpression, we analyzed cell death and the Bax oligomeric state stabilized by the cross‐linker EGS (Fig. 6D). It has been proposed that Bax homo‐oligomerization at the OMM leads to the release of apoptogenic factors [6]. Etoposide treatment induced Bax overexpression following a 48‐h incubation. However, Bax levels were similarly increased following a 70‐h incubation with and without etoposide, yet Bax dimers were observed only in the presence of etoposide. Moreover, although Bax levels were similar following incubation with etoposide for 48 and 70 h, Bax dimers were only detected after 70‐h incubation with etoposide. However, cell death (Fig. 6D, bottom) was not dependent on Bax dimers, as about 80% was observed at 48 h with no Bax dimers, and about 90% at the 70‐h incubation with etoposide when Bax dimers were present. Thus, Bax dimerization does not seem to be responsible for cell death.

Next, we tested the effects of silencing Bax expression using Bax‐specific siRNA (si‐Bax) on etoposide‐induced cell death (Fig. 6E–H). si‐Bax at various concentrations showed a reduction in Bax levels, with up to a 90% decrease achieved at a concentration of 75 nm of si‐Bax (Fig. 6E,F). This reduction in Bax expression levels did not correlate with a decrease in etoposide‐induced apoptosis. Even with over 90% reduction in Bax level was reduced (Fig. 6H), only a 10% decrease in cell death was obtained (Fig. 6G)

The expression levels of VDAC1, VDAC1‐ΔC, p53, and p21 were monitored in cells treated with si‐Bax, or with nontargeted siRNA (si‐NT) and thereafter incubation for 48 h with etoposide (Fig. 6H). Interestingly, etoposide treatment of cells silenced for Bax expression had no effect on the VDAC1, VDAC1‐ΔC, and p53 expression levels, but resulted in a reduction in p21 expression levels (Fig. 6H).

The decrease in p21 levels, although with no change in p53, is surprising as p21 expression is dependent on wild‐type p53. However, p21 can mediate cell‐cycle arrest at G1 phases through p53‐dependent or independent pathways, and p21 expression is increased through p53‐dependent and p53‐independent pathways [47], and induction of p21 expression by DNA‐damaging agents does require p53 [48]. For instance, in gastric cancers, no apparent correlation was noted between p21 and p53 expression [49].

### 3.6 Etoposide‐induced VDAC1‐ΔC formation and cell death is dependent on cell type and the Topoisomerase inhibitor

**Fig. 7:**
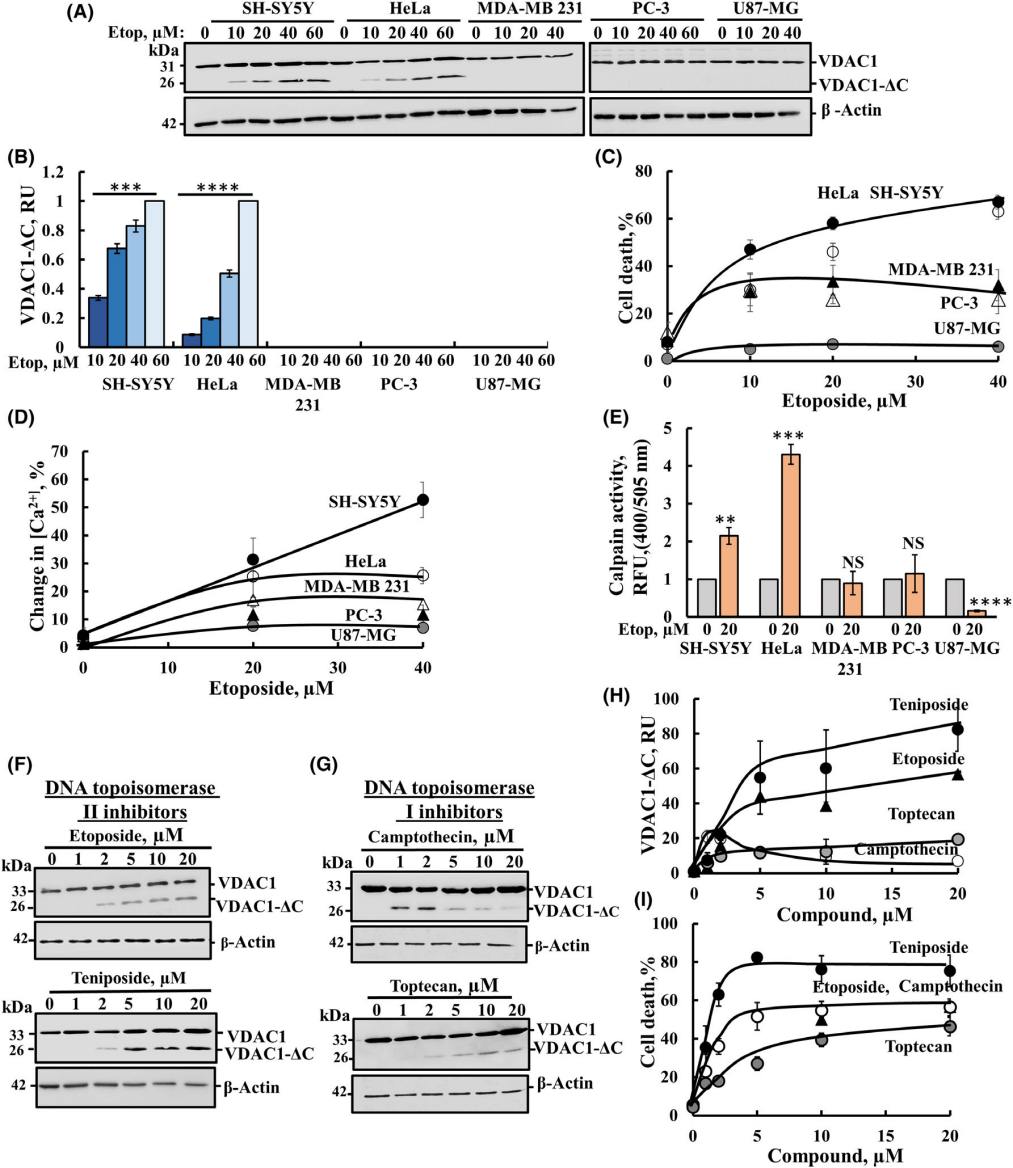
Etoposide‐induced VDAC1 truncation and cell death is dependent on cell type and the Topo‐I and Topo‐II inhibitors used. (A–C) The indicated different cell lines were incubated with different concentrations of etoposide for 48 h and then immunoblotted for VDAC1 and VDAC1‐ΔC (A), and VDAC1‐ΔC levels were quantified and presented in relative units (RU) (B), and cell death was analyzed using PI staining and FACS analysis (C). (D, E) The indicated cell lines were incubated with different concentrations of etoposide for 16 h, and then analyzed for cytosolic [Ca^2+^] using Fluo‐4 and FACS analysis (D) and for calpain activity, represented in Relative Fluorescence Unit (RFU) (E). (F–I) SH‐SY5Y cells were incubated for 48 h with different concentrations of the Topo‐II inhibitors, etoposide and teniposide (F) or the Topo‐I inhibitors, camptothecin and topotecan (G), and the levels of VDAC1‐ΔC were analyzed by immunoblotting (F, G). Their quantification is presented in relative units (RU) (H), and cells were assayed for cell death using propidium iodide staining FACS analysis (I). The significance of the differences between the control and treated sample was determined using Student's *t*‐test, conducted with the *t*‐test function in Microsoft Excel. Results are the means ± SEM (*n* = 3). **P ≤ 0.01, ***P ≤ 0.001, ****P ≤ 0.0001. NS, nonsignificant.

The activity of etoposide in producing VDAC1‐ΔC and inducing cell death was tested in several cell lines other than SH‐SY5Y, including HeLa, MDA‐MB‐231, PC‐3, and U‐87MG, derived from cervical, breast cancer, prostate cancer, and brain cancer, respectively (Fig. 7). The results show that etoposide treatment resulted in the formation of VDAC1‐ΔC in SH‐SY5Y and HeLa cells, but not in the other tested cell lines (Fig. 7A,B). These cell lines also exhibited the highest levels of etoposide‐induced cell death, reaching about 80% and 70%, respectively, while about 30% cell death were obtained with PC‐3 and MDA‐MB ‐231 and no cell death with U‐87MG cells (Fig. 7C). The results suggest a correlation between etoposide‐mediated VDAC1‐ΔC formation and cell death induction.

To identify possible reasons for the differential effect of etoposide in the different cell lines, we analyzed its effects on the expression levels of three apoptosis‐related proteins: Bax, p53, and p21, on intracellular Ca^2+^ levels and on calpain activity. Etoposide, in a concentration‐dependent manner, increased the expression of the apoptosis‐related proteins in all tested cell lines (Fig. S2A–C). However, while etoposide increased intracellular Ca^2+^ in HeLa and SH‐SY5Y, and to a lesser extent in PC‐3 and MDA‐MB 231, no increase was observed in U87‐MG (Fig. 7D). Similarly, calpain activity was obtained in HeLa and SH‐SY5Y, with some activity in PC‐3 and MDA‐MB 231, but not in the U87‐MG (Fig. 7E). These results suggest that VDAC1‐ΔC was not formed in some cell lines because etoposide did not increase cytosolic [Ca^2+^] and, subsequently, activation of calpain differed among the cell lines.

### 3.7 The activity of different DNA topoisomerase inhibitors on cell death and VDAC1 truncation

Next, we tested, in addition to etoposide, another Topo‐II inhibitor, teniposide, along with Topo‐I inhibitors, camptothecin and topotecan, on VDAC1‐ΔC formation and cell death induction (Fig. 7F–I). SH‐SY5Y cells were incubated with different concentrations of each of the four Topo‐I or Topo‐II inhibitors. The results showed that Topo‐II inhibitors induced higher levels of VDAC1‐ΔC (Fig. 7F,H) compared to Topo‐I inhibitors (Fig. 7G,H). Similarly, topo‐II inhibitors induced higher cell death, than Topo‐I inhibitors (Fig. 7I).

## 4 Discussion

### 4.1 Etoposide and cell death induction

The ability of topoisomerases to introduce single (type I) or double (type II) DNA breaks (Topo‐I and Topo‐II, respectively) and then repairing the damage is essential for correct DNA replication, transcription, chromosome segregation, and recombination. Inhibiting the reannealing stage by specific inhibitors, which form a Topo‐DNA‐inhibitor complex, is a powerful way for inducing apoptosis. The improperly repaired DNA initiates cell death via activation of the intrinsic pathway [50, 51], which involves the induction of poly (ADP‐ribose) polymerase‐1 (PARP‐1) expression, the release of pro‐apoptotic molecules from mitochondria, and the activation of cytoplasmic caspases. Topo‐mediated DNA damage activates DNA sensors, including the kinases ATM (Ataxia Telangiectasia Mutated), ATR (Ataxia Telangiectasia and Rad 3‐related), and DNA‐PK (DNA‐dependent protein kinase) which phosphorylate a large number of pro‐apoptotic substrates, such as p53 and Chk2, leading to apoptosis activation [52]. Additionally, in some cell types, the extrinsic pathway, involving Fas‐associated death domain (FADD) and caspase‐8, is also activated [52, 53]. Due to this powerful apoptotic induction, a variety of topoisomerase inhibitors have been long utilized as anticancer drugs.

**Fig. 8:**
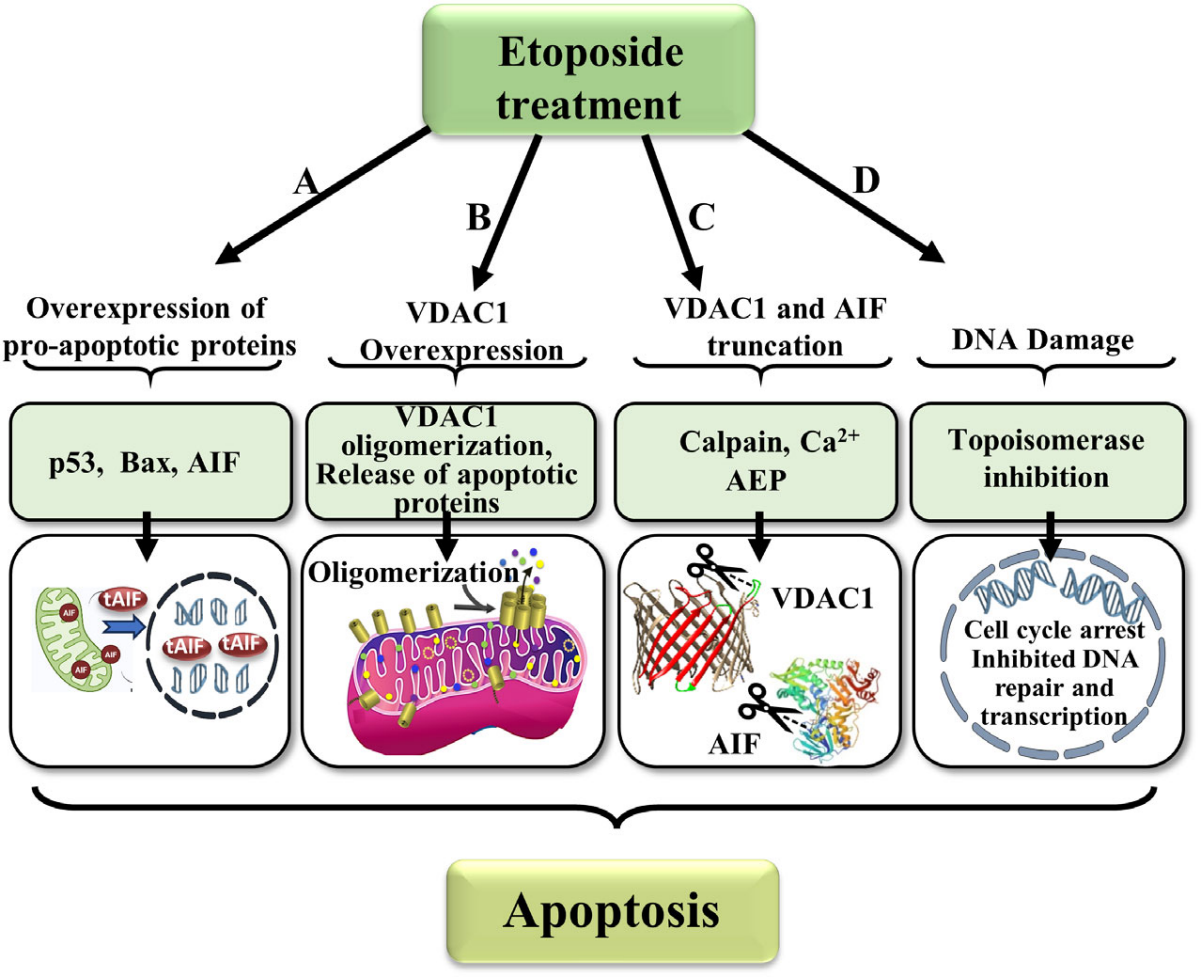
Proposed molecular mechanisms for etoposide mode of action associated with cell death induction. Cells treated with etoposide resulted in multiple effects leading to apoptotic cell death. These included: (A) Overexpression of p53, Bax, and AIF. The low contribution of Bax to cell death was demonstrated through silencing its expression, which only slightly affected cell death. AIF increased levels, and its cleavage by calpain produced truncated‐AIF (tAIF) that is released from the mitochondria via the VDAC1 oligomer, followed by its translocation to the nucleus, contributing to caspase‐independent apoptosis. (B) Etoposide‐induced overexpression of VDAC1 that may result from the increase in mitochondrial ROS and p53 levels induced by etoposide, both of which contributed to the overexpression of VDAC1 [38]. This leads to its oligomerization, release of pro‐apoptotic proteins and, thus, apoptosis activation. (C) Etoposide induced increased AEP and calpain expression levels, with the elevated [Ca^2+^]i, leading to calpain activation and upregulation of AEP. Active calpain cleaved VDAC1 to produce VDAC1‐ΔC. This is inhibited by calpain and the AEP inhibitor, and the Ca^2+^ chelator BAPTA‐AM. (D) Etoposide induced DNA damage, leading to sensor molecule activation and recruitment of signal transducer proteins, causing effector molecule function to temporarily arrest the cell cycle, transcribe DNA repair proteins, and repair the site of damage. If repair cannot be achieved, apoptosis is activated. Thus, etoposide possesses multiple activities, and not only induces DNA damage by inhibiting Topo‐II.

In this study, we explored additional mechanisms by which the Topo‐II inhibitor, etoposide, induces cell death (Fig. 8).

### 4.2 Etoposide‐induced cell death is associated with VDAC1 overexpression and oligomerization

Etoposide‐induced DNA damage has been previously shown to activate p53 and upregulate the pro‐apoptotic protein Bax [54].

The tumor suppressor protein p53 plays a central role in the regulation of the cell cycle, apoptosis, and senescence in response to a broad range of stresses, functioning through both transcription‐dependent and transcription‐independent mechanisms [55, 56, 57]. Our results confirm that p53 levels are increased following etoposide treatment. However, despite similar p53 levels after 24 and 70 h incubation, cell death was significantly lower at 24 h (Figs. 1 and 6A). Therefore, p53 alone cannot account for the regulating of etoposide‐induced apoptosis.

One possibility is that etoposide‐activated p53 is responsible for Bax overexpression [54, 58] (Fig. 6A) forming large channels composed of Bax and/or Bak oligomers that mediates the release of pro‐apoptotic proteins [59]. However, this mechanism appears unlikely in our case, as silencing Bax expression had no effect on the extent of cell death induced by etoposide (Fig. 6G,H).

Our results propose an additional mechanism (Fig. 8B), by which etoposide induces overexpression of VDAC1, shifting the equilibrium from monomeric to oligomeric VDAC1. This VDAC1 oligomerization forms a large channel that mediates the release of apoptogenic proteins, leading to apoptosis (Figs. 2A,B and 8B). The upregulation of VDAC1 overexpression is induced by ROS, [Ca^2+^]i [18, 19, 60, 61], and p53, all of which are induced by etoposide [54, 58]. Specifically, p53 has been shown to modulate VDAC1 expression and oligomerization [62, 63]. Similar to various apoptosis inducers and stress conditions, such as curcumin, As_2_O_3_, cisplatin, selenite, H_2_O_2_, or UV light, etoposide induces the overexpression and oligomerization of VDAC1, a key step in the release of pro‐apoptotic proteins from the IMS to the cytosol [11, 12, 13, 15, 16, 17, 18, 19, 20, 27, 28, 41, 64, 65].

### 4.3 Etoposide‐induced overexpression calpain and AEP to truncate VDAC1 and AIF

Here, we present, for the first time that etoposide induces C terminus truncation of VDAC1 (Figs 2, 4, and 6), a phenomenon previously observed under hypoxic conditions [29, 30, 31]. Interestingly, both conditions share a common mechanism for VDAC1‐ΔC formation, requiring Ca^2+^, calpain, and the lysosomal AEP proteases catalyze the formation of VDAC1‐ΔC (Figs 3 and 4, Fig. S1) [29, 30, 31] As a corollary, etoposide‐induced VDAC1‐ΔC formation and cell death were completely prevented by a calpain‐1‐specific inhibitor and the Ca^2+^ chelating reagent, BAPTA‐AM (Figs 4 and 8C).

Calpain‐1 and calpain‐2 are calcium‐activated cysteine proteases that cleave a variety of proteins and participate in a wide range of cellular processes, including transcription, survival, proliferation, apoptosis, migration, and invasion. The observation that activation of calpain can activate apoptotic and other signaling pathways [66], makes it a potential therapeutic target in diseases, including Alzheimer's disease and several types of cancer [67]. Indeed, dysregulated calpain activity has been implicated in tumorigenesis and cancer progression [68]. Upregulation of calpain‐1 and calpain‐2 have also been described in several cancers [69] and high calpain‐1 expression has been associated with a shorter disease‐free survival in breast cancer patients treated with trastuzumab [70]. The link between elevated calpain expression and poor prognosis is attributed to promotion of cellular survival and cytoskeletal remodeling, fostering a more invasive phenotype. It may be important not to forget this. Our finding of etoposide‐induced increased calpain expression levels underscores the importance of considering the association between increased calpain expression and a poor prognosis of certain cancer types when recommending this type of therapeutic approach [70].

Importantly, calpains are involved in apoptosis through various mechanisms. Emerging evidence suggests that several chemotherapy agents can modulate calpain activity, thus influencing apoptosis. Specifically, calpain can cleave the autophagy‐related gene (Atg) 5 protein, which is an important factor in drug‐induced apoptosis [71]. Similarly, both calpain‐1 and calpain‐2 are activated by paclitaxel treatment in non‐small cell lung carcinoma cells, contributing to paclitaxel‐mediated cell death [72].

Our results reveal that etoposide induces overexpression of calpain, and inhibition of calpain activity by a specific inhibitor reduces cell death (Fig. 4A–F). This effect may be a consequence of inhibition of VDAC1‐ΔC formation and/or the activation of AIF, which induces chromatin condensation and DNA fragmentation. Calpain is required to produce and release truncated‐AIF (tAIF) from the mitochondria and thus facilitated its translocation to the nucleus (Fig. 5). In the nucleus, tAIF activates caspase‐independent cell death pathways (Fig. 8C,D).

Mammalian AEPs, which are predominantly localized in late endosomes and lysosomes, selectively cleave peptide bonds bearing Asn or Asp residues [73]. Our results indicate that etoposide enhances the expression of AEP, particularly of the mature form (Fig. S1B–E), possibly because the etoposide‐induced elevation of cellular Ca^2+^, which activates the AEP promoter [45]. The role of AEP in catalyzing VDAC1 truncation is supported by the inhibition of VDAC1‐ΔC formation by a specific AEP inhibitor (Fig. 8C, Fig. S1A–E).

The expression levels of AEP regulate cell proliferation [45], with high levels of AEP being linked to poorly differentiated tumors and increased necrosis and apoptosis [45]. A Kaplan–Meier analysis of AEP expression levels in 99 cases of glioblastoma multiforme (GBM) reveals that patients with low AEP expression had a median survival time of 26 months whereas those with high AEP expression had a median survival time of 8 months [43]. Hence, it is plausible to establish a connection between resistance to etoposide and the elevated AEP levels described in this study.

### 4.4 The relation between VDAC1 truncation and cell death

VDAC1 cleavage does not release any fragment, as the protein is embedded in the membrane and the cleaved protein when purified includes both the 26 kDa and the C terminus fragment [29]. Consequently, although the interaction sites between the VDAC1 monomers in the oligomeric structure involve β‐strands 8 and 16 [28], we observed no effect of the cleavage on VDAC1 oligomerization.

The relationship between VDAC1‐ΔC formation and cell death was examined via several approaches:

- Inhibition of etoposide‐induced VDAC1‐ΔC formation by specific inhibitors of calpain or AEP also inhibited cell death (Fig. 4A–D). In addition, the increases in both cell death and VDAC1‐ΔC formation in etoposide‐treated cells were inhibited by the cell‐permeable Ca^2+^ chelating reagent, BAPTA‐AM (Figs 3E and 4G,H), suggesting a link between VDAC1 truncation and the elevation of cytosolic Ca^2+^.
- In different cell types, a correlation was found between etoposide‐induced VDAC1 truncation and cell death induction (Fig. 7) Specifically, etoposide induced high levels of VDAC1‐ΔC formation and cell death in SH‐SY5Y and HeLa cells, while low effect was observed in PC‐3, MDA‐MB 231, and U87‐MG cell lines. This seems to be connected to the etoposide‐induced high elevation of intracellular Ca^2+^ in HeLa and SH‐SY5Y cells, compared to a moderate elevation of [Ca^2+^]i in PC‐3, MDA‐MB 231 cells, and no increase in U87‐MG cells. This lack of increase [Ca^2+^]i by etoposide validates the linkage between etoposide activation of the Ca^2+^‐dependent calpain, and the formation of VDAC1‐ΔC and subsequent cell death.
- A comparison of the effects of inhibitors of Topo‐II (etoposide and teniposide) and Topo‐I (camptothecin and topotecan) support the link between VDAC1‐ΔC formation and cell death (Fig. 7F–I). Topo‐II inhibitors inducing more VDAC1‐ΔC and cell death than Topo‐I inhibitors.

The relationship between the formation of VDAC1 truncation and apoptosis induction as induced by etoposide is not clear—whether VDAC1‐ΔC induces apoptosis, or apoptosis induces its formation.

To address the relationship between the two processes, in our previous study, we constructed and expressed VDAC1‐ΔC in cell and found that it is not targeted to the mitochondria and accumulated in the cell as insoluble protein [29]. However, the two processes are tightly linked: they show the same concentration‐depends, both are inhibited by calpain and AEP or reduced in the absence of intracellular Ca^2+^ at the same time‐ and concentration dependency.

The correlation between the extent of VDAC1‐ΔC formation and the induction of cell death presented above underscores the potential link between VDAC1 truncation and apoptotic induction. Interestingly, VDAC1‐ΔC has been detected in advanced stage of non‐small cell lung carcinoma patients and may be associated with resistance to etoposide [32]. It should be noted, however, although the VDAC1‐ΔC formation induced by hypoxia and etoposide involve similar mechanism, in hypoxia, the hypoxia‐inducible factor 1A (HIF‐1α) is required for the hypoxia‐induced VDAC1 truncation but not for etoposide‐induced VDAC1 truncation (Fig. S2F).

### 4.5 Molecular mechanism of etoposide: beyond topoisomerase inhibition

Besides the well‐known activity of the Topo‐II inhibitor, recent studies suggest that etoposide‐triggered activation of ATM may promote mitochondrial biogenesis and regulate ROS production [74]. In addition, etoposide has been observed to disrupt mitochondrial function within the cell and deplete the ATP pool [74].

Our results, as summarized above and in Fig. 8, reveal that etoposide‐induced cell death extends beyond the inhibition of Topo‐II, correlating with the activation of mitochondrial apoptotic pathways. These effects encompass: (A) overexpression of apoptotic regulators such as p53, Bax, and AIF; (B) induction of VDAC1 overexpression leading to its oligomerization, subsequent release of pro‐apoptotic proteins and, thus, apoptosis activation; (C) Etoposide, induced increases expression levels of AEP and calpain along with elevated [Ca^2+^]i, mediating VDAC1‐ΔC formation.

Increased levels of [Ca^2+^]i, ROS, and p53 can lead to VDAC1 overexpression [18, 19, 60, 61, 62, 63] and subsequent cell death [11, 12, 13, 15, 16, 17, 18, 19, 20, 27, 28, 41, 64, 65, 75, 76, 77].

These findings provide a more comprehensive understanding of etoposide action at the molecular level and its diverse effects on cellular processes, which are associated with cell death activation, emphasizing its effects beyond Topo‐II inhibition (Fig. 8).

### 4.6 Clinical application of etoposide: key considerations

Topo‐I and Topo‐II inhibitors have attracted much interest as therapeutic agents against infectious and cancerous cells and are listed by the World Health Organization as essential medicines. Etoposide is extensively employed in the treatment of various cancers. Currently, the major known mechanisms of etoposide resistance include altered expression of Topo‐II, decreased expression of genes involved in DNA mismatch repair, and increased expression of ABC transporters, such as MDR1 and MRP1 [78]. However, sensitivity to etoposide is not solely determined by the levels of topoisomerases present in the cells, and the additional mechanism for etoposide‐induced cell death presented here should also be considered. Etoposide induced overexpression and oligomerization of VDAC1 associated with cell death (Fig. 1) and increased levels of calpain (Fig. 4E,F) and AEP (Fig. S1B–E), and its cancer cell type‐dependent effect (Fig. 7E). Importantly, the findings presented here provide a foundation for potential developments toward precision medicine for cancer treatment, and the modes of etoposide actions and resistance that should be considered.

## 5 Conclusions

This study elucidates the complex mechanisms by which the DNA topoisomerase inhibitor etoposide induces cell death. Etoposide treatment results in both the overexpression and truncation of VDAC1, which are closely associated with apoptotic cell death. Additionally, etoposide increases the expression of calpain and asparagine endopeptidase, enzymes that mediate VDAC1 truncation. Specific inhibitors of these enzymes not only prevent VDAC1 truncation but also abolish etoposide‐induced cell death. The levels and activity of calpain in various cell lines correlate with their sensitivity to etoposide, suggesting a potential link to drug resistance. These findings shed light on the variable outcomes of etoposide treatment observed in clinical settings and offer new insights into strategies for predicting resistance and optimizing its use.

## Conflict of interest

The authors declare no conflict of interest.

## Author contributions

AKN carried out the experiments, data analysis, and figure preparation. VS‐B designed the experimental strategy, interpreted the results, arranged the figure presentation, and wrote the paper.

## Supporting information


**Fig. S1.** Etoposide‐induced VDAC1 truncation is inhibited by VBIT‐4 and AEP inhibitor‐I and etoposide increased AEP expression.
**Fig. S2**. Etoposide induces increased expression levels of Bax, p53, and p21 in different cell types.
**Table S1**. Antibodies used in this study.

## Data Availability

All data reported in this paper will be shared by the lead contact (vardasb@bgu.ac.il) upon request. Any additional information required to reanalyze the data reported in this paper is available from the lead contact upon request.
