# Supplementary material for: Etoposide‐induced cancer cell death: roles of mitochondrial VDAC1 and calpain, and resistance mechanisms
Source: Mol Oncol. 2025 Feb 7;19(6):1855–75. doi: 10.1002/1878-0261.13807 (PMC12161478; doi:10.1002/1878-0261.13807)
Supplement: Supplementary file 1 — Fig. S1. Etoposide‐induced VDAC1 truncation is inhibited by VBIT‐4 and AEP inhibitor‐I and etoposide increased AEP expression. Fig. S2. Etoposide induces increased expression levels of Bax, p53, and p21 in different cell types. Table S1. Antibodies used in this study. [file MOL2-19-1855-s001.pdf]

Supplementary Materials

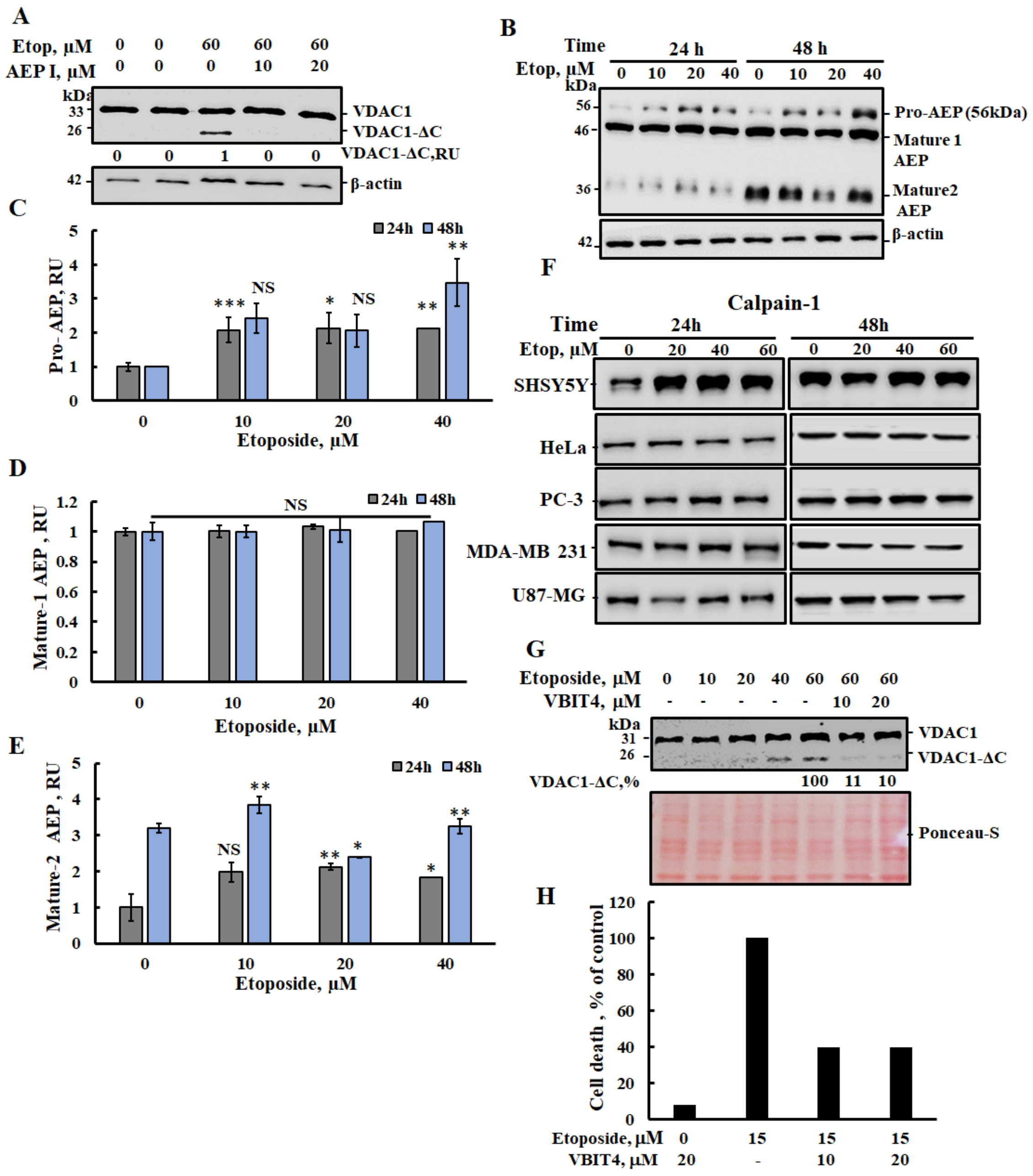

Fig. S1. Etoposide-induced VDAC1 trunction is inhibited by VBIT-4 and AEP inhibitor-I and etoposide increased AEP expression

(A) SH-SY5Y cells were incubated with or without 60 µM etoposide for 48h in the absence and presence of the indicated concentration of AEP inhibitor-I. VDAC1-ΔC formation was analyzed by immunoblotting. (B–E) SH-SY5Y cells were incubated with different concentrations of etoposide for 24h and 48h, and the levels of pro- and mature-1 and mature-2 AEP were analyzed by immunoblotting with specific antibodies, and their levels were quantified and presented in relative units (RUs) (C, D,E). (F) SH-SY5Y, HeLa, PC-3, MDA-MB 231, and U87-MG cells were incubated with different concentrations of etoposide for 24h and 48 h, and the levels of calpain1 were analyzed by immunoblotting with specific antibodies. (G,H) Hela cells were pre-incubated with VBIT-4 (10 and 20 µM, 2h). The cells were then incubated for 48h with and without the indicated concentrations of etoposide and subjected to immunoblotting using anti-VDAC1 antibodies (G) and to cell death analysis (H). The expression levels of VDAC1-ΔC were quantified and presented as the % of the control (without VBIT-4) at the bottom of the blot.

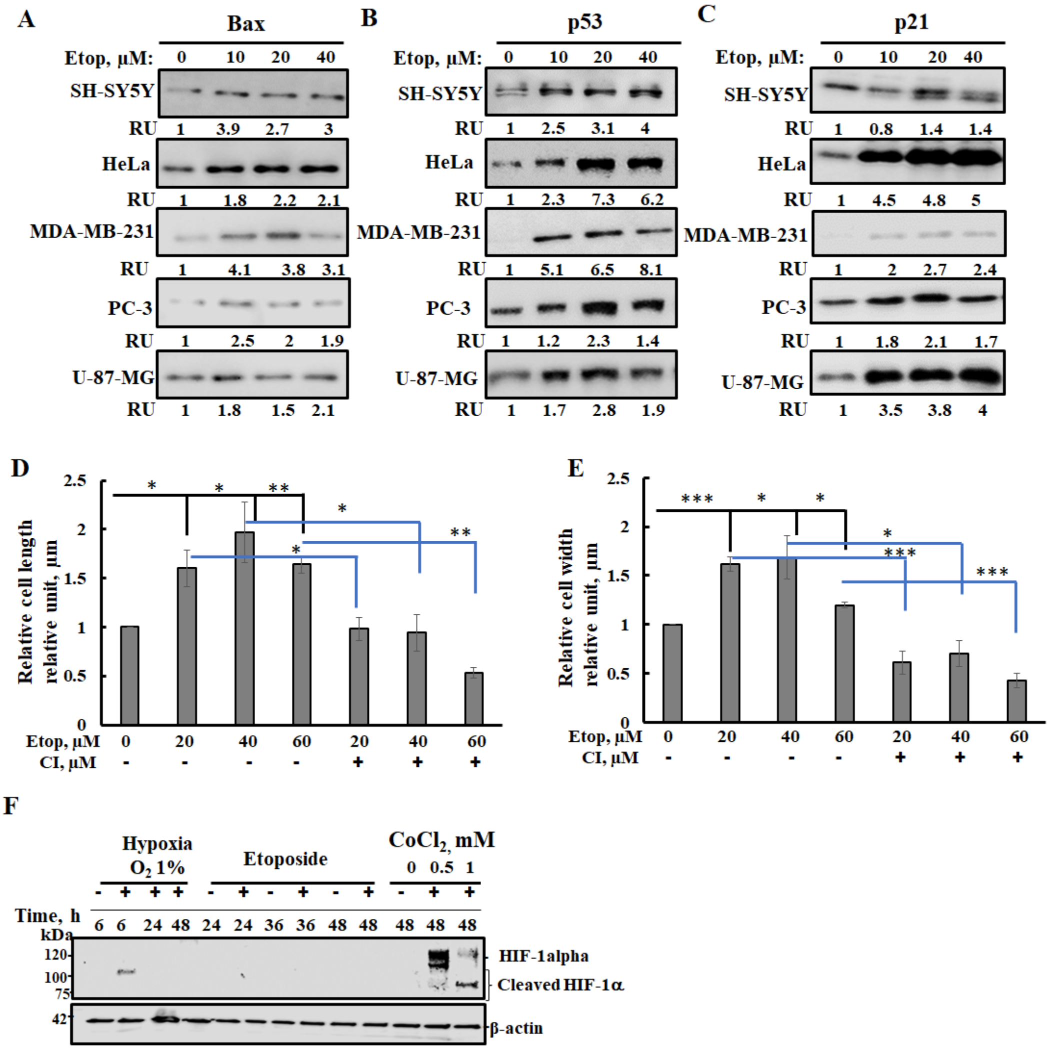

Fig. S2. Etoposide induces increased expression levels of Bax, p53, and p21 in different cell types

(A–C) The indicated cell lines were incubated with different concentrations of etoposide for 48h and immunoblotted using specific antibodies against Bax, p53, and p21. The protein relative expression levels were quantified and presented as relative units (RUs) at the bottom of each blot. (D-E) Quantitative analysis of relative cell size, length and width of SH-SY5Y cells incubated with the indicated etoposide concentrations in the absence or first pre-incubated with CI (Calpain Inhibitor) (4 µM, 1h) followed by 48h. (F) SH-SY5Y cells were incubated with different indicated conditions and immunoblotted using Hif-1α antibody. First 4 lanes indicates cells with or without incubated in 1% O_2, following lanes indicates cells were incubated with or without 40 µM etoposide for 24h, 36h, or 48h , and the last 3 lanes represents cells incubated with or without $CoCl_2$ (0.5 and 1 mM) for 48h.

Table S1. Antibodies used in this study

Antibodies against the indicated protein, their catalogue number, source, and the dilutions used in immunoblot, and immunofluorescence experiments are presented.

Antibody	Source and Cat. No.	WB	IF
Rabbit monoclonal anti-VDAC1(against the N-terminal region)	Abcam, Cambridge, UK, ab154856	1:5000	-
Rabbit monoclonal anti-VDAC1 (150-250 AA-targeted)	Abcam, Cambridge, UK, Ab15895	1:5000	-
Rabbit polyclonal Anti-calpain-1	Abcam, Cambridge, UK, ab28258	1-8000	-
Rabbit monoclonal anti-AIF-1	R&D Systems, Minneapolis, AF1457	1:2000	1:500
Rabbit polyclonal anti-Bax	Abcam, Cambridge, UK, ab53154	1:1000	-
Rabbit monoclonal anti-p53	Abcam, Cambridge, UK, ab32049	1:1000	
Rabbit monoclonal anti- p21	Abcam, Cambridge, UK, ab109520	1:1000	-
Rabbit monoclonal anti-AEP	Abcam, Cambridge, UK, ab183028	1:20000	-
Mouse monoclonal anti-Actin	Milipore, Billerica, MA, MAB1501	1:10000	-
Phlloidin-iFluor 488 Reagent	Abcam, Cambridge, UK, ab176753	-	1:1000
Anti-Rabbit IgG, HRP conjugate	Promega Corporation, WI, USA.	1:10000	-
Anti-Mouse IgG, HRP conjugate	Abcam, Cambridge, UK, ab98799	1:10000	
Anti-Rabbit IgG, Alexa Fluor 555	Abcam, Cambridge, UK, ab150086	-	1:1000
